# Antiproliferative and Proapoptotic Effects of *Labisia pumila* Ethanol Extract and Its Active Fraction in Human Melanoma HM3KO Cells

**DOI:** 10.1155/2012/123470

**Published:** 2012-03-08

**Authors:** Azimahtol Hawariah Lope Pihie, Zainul Amiruddin Zakaria, Fezah Othman

**Affiliations:** ^1^School of Biosciences and Biotechnology, Faculty of Science and Technology, National University of Malaysia, 43600 Bangi, Malaysia; ^2^Department of Biomedical Sciences, Faculty of Medicine and Health Sciences, Universiti Putra Malaysia, 43400 Serdang, Malaysia

## Abstract

The present study was to determine the anticancer potential of *Labisia pumila* in *in vitro* models. Results from the study revealed that ethanol extract of *L. pumila* was more cytotoxic against HM3KO cells while having reduced effects on nonmalignant cells as compared to aqueous and hexane extracts. Thus, ethanol extract was selected to be further separated by using the bioassay-guided fractionation method to give an active fraction, SF2Lp. Results obtained from the flow cytometry analysis showed that SF2Lp was able to arrest the HM3KO cell cycle at the G1 phase, while morphological findings from AO-EB nuclear staining assays along with the Apoptotic Index confirmed the induction of apoptosis by SF2Lp in HM3KO cells. Results from the mechanistic study further revealed that SF2Lp treatment was able to concurrently increase the expression level of p53 and pro-apoptotic protein Bax and also reduce the expression level of anti-apoptotic protein BCl-2 in HM3KO cells, directly contributing to the increase in Bax/Bcl-2 ratio. These findings, therefore, suggested that *L. pumila* was able to inhibit HM3KO cell growth possibly by arresting the cell cycle at G1 phase and inducing apoptosis in HM3KO cells via the up- and down-regulation of Bax/Bcl-2 protein, mediated through a p53-dependent pathway.

## 1. Introduction

Natural products of various sources, particularly from plants and marines have been regarded as a precious alternative to modern medicine and investigations on active components with anticancer potential of natural sources have been extensively carried out [1–4].

There is an increasing understanding that chemotherapeutic agents and a variety of anticancer agents can stimulate cancer cell death by way of apoptosis [5–8]. Apoptosis, a highly structured and orchestrated process, performs a significant role in regulating cell number for the growth and homeostasis of tissues by eliminating aged, damaged, and unwanted cells [9, 10]. In cancer treatment, one of the approaches to restrain tumor growth is by activating the apoptotic machinery in the tumor cells [11, 12]. 

Earlier studies done revealed that extracts from the plants of Myrsinaceae exhibited anticancer potential in both *in vitro* or *in vivo* models [13–15]. *Labisia pumila* (*L. pumila*), or locally recognized as Kacip Fatimah from the family Myrsinaceae, is a famous Malaysian traditional herbs that has been exploited especially by the Malay women for generations for pre- and postpartum treatments [16, 17]. Other applications of *L. pumila* include treatment of dysentery, dysmenorrhea, flatulence, gonorrhea, and “sickness in the bones” [16, 18]. Of late, the herb has been extensively commercialized in Malaysia as health tonic drink and supplement capsules especially for women.

Scientific studies done on *L. pumila* were very scarce and published data on the pharmacological activity of this plant were very limited. Several scientific studies done on *L. pumila *revealed that *L. pumila *aqueous extract was found to have compounds with oestrogenic activities [19] and was able to reduce the level of cortisol in pregnant lab rats without affecting the status of the immune system [20]. In a different study, the water extract of this plant demonstrated protective effect on UV-irradiated human dermal fibroblasts, and the effect was found better than ascorbic acid in defending the skin against UV-induced photoaging [21]. Besides these reports, published scientific findings regarding anticancer potential of *L. pumila in vitro* have not yet been reported. Thus, this study was intended to investigate the antiproliferative potential of *L. pumila* ethanol extract and its active fraction in *in vitro* model and also to determine the molecular mechanism involved during the induction of apoptosis in human melanoma HM3KO cells. To the best of our knowledge, this is the first information on the antiproliferative and proapoptotic effects of *L. pumila* in human melanoma HM3KO cells* in vitro*.

## 2. Materials and Methods

### 2.1. Plant Material

Dried powder of *L. pumila* whole plant was supplied by Professor Dr. Azimahtol Hawariah Lope Pihie (National University of Malaysia).

### 2.2. Plant Extraction

In this study, the dry powder of *L. pumila* whole plant was separately extracted with hexane, ethanol, and water. For the preparation of ethanol and hexane extracts, *L. pumila* whole plant powder was weighed and exhaustively extracted with 90% ethanol and absolute hexane (1 g/10 mL, w/v), respectively, by using a Soxhlet apparatus at a temperature of 40–50°C for 8 hours. The extracts obtained were then filtered through No. 2 Whatman filter paper and both filtrates were dried at 40°C under reduced pressure by using a rotary evaporator. As for the aqueous extract, it was prepared by heating *L. pumila* whole plant powder with distilled water (1 g/10 mL, w/v) at a temperature of 60°C for 8 hours. The resultant extract was then filtered through No. 2 Whatman filter paper, and the filtrate was freeze-dried by using a freeze-dryer.

### 2.3. Preparation of Samples

To determine the antiproliferative activity of *L. pumila*, all dried hexane (LpHE), ethanol (LpEE), and aqueous (LpAE) extracts were weighed and dissolved in dimethyl sulphoxide (DMSO) to an appropriate concentration and stored as a stock solution at −20°C until used. The final concentration of DMSO used was less than 1%, and at this concentration, DMSO does not affect cell viability [22]. For the treatment of experimental cultures, each stock of extracts was diluted with fresh Dulbecco's Modified Eagle's Medium (DMEM) enriched with 10% (v/v) fetal bovine serum (FBS) and 1% antibiotic (Penicillin and Streptomycin) cocktail to give final concentrations of *L. pumila* extracts ranging from 0 to 5 mg/mL.

Active fraction of *L. pumila* was prepared from the most active extract by using column chromatography, where chloroform with an increasing amount of methanol was used as the eluent. Fractions collected were then subjected to thin layer chromatography (TLC) using methanol : chloroform (1 : 9) as the mobile phase. Fractions with the same TLC profile were pooled and dried to give a few main fractions. The fraction with most yield was then chosen for further separation and rechromatographed and fractions collected were then underwent TLC profiling to give main fractions. These fractions were then subjected to antiproliferative assay against HM3KO cells to select the most active fraction. The selected active fraction was then diluted in DMSO to generate various concentrations based on its IC_50_ value, to be further investigated in the apoptosis assay, cell cycle progression, and Western blot analyses.

### 2.4. Chemicals

Dacarbazine or 5-(3,3-Dimethyl-1-triazenyl)imidazole-4-carboxamide or DTIC, ethylenediamine tetraacetic acid (EDTA), ribonuclease A (RNase A), dimethyl sulfoxide (DMSO), proteinase K, acridine orange, ethidium bromide, and propidium iodide were purchased from Sigma Chemical Co (St. Louis, MO, USA). Dulbecco's modified Eagle's Medium (DMEM), trypsin, fetal bovine serum (FBS), EDTA, and penicillin-streptomycin were purchased from Gibco Laboratories, New York, while fungizone was bought from Flowlab, Australia.

The antibodies against Bax (clone 6A7), Bcl-2 (clone Bc-12/100), and p53 (clone Pab 1801) were purchased from Pharmingen (USA). The antibody against *β* actin was from Sigma Aldrich. Bradford reagent was from Bio-Rad laboratories (USA) and Renaissance Western blot Chemiluminescence reagent Plus was from Perkin Elmer (Boston, USA). All other chemicals used in this study were of the highest grade available.

### 2.5. Cell Culture

Human melanoma HM3KO cells were kindly provided by Dr Yoko Funasaka, Japan, whereas MDBK and Vero cells were purchased from American Type Cell Culture Collection (ATCC), Maryland, USA. These cells were cultured in DMEM supplemented with 10% fetal bovine serum, penicillin-streptomycin, fungizone, and miramycin. The cells were maintained in a humidified incubator at 37°C with 5% CO_2_ and 95% air. The cells were regularly observed using an inverted microscope. 

### 2.6. Cell Proliferation Assay

The antiproliferative effects of *L. pumila* various extracts (hexane-LpHE, ethanol-LpEE, and aqueous-LpAE) were investigated by determining their IC_50_ values. Cells were cultured in supplemented DMEM in a humidified atmosphere with 5% CO_2_ at 37°C. When the cells reached 70–80% confluency, these logarithmically growing cells were then rinsed with phosphate buffered saline (PBS) before being trypsinized with 0.025% trypsin. Cells (1 × 10^5^) were then plated in a 96-well plate and permitted to adhere for 8–12 h. On the following day, old media were discarded and all cells were rinsed with PBS. Fresh supplemented DMEM was then loaded into each well and cells were then treated with various concentrations (0–5 mg/mL) of LpAE, LpEE, and LpHE, 1% DMSO (served as negative control), and Dacarbazine (positive control). Dacarbazine was chosen to be used as positive control because it is one of the most active approved neoplastic agents for the treatment of malignant melanoma [23]. The treated and untreated cells were then incubated for 24, 48, and 72 hours at 37°C in an atmosphere of 5% CO_2_ and 95% air. At the end of each indicated time, the antiproliferative activity of all extracts was assessed by using methylene blue method as previously described [24]. The absorbance of dissolved solutions was measured by an ELISA reader at a wavelength of 660 nm. Each concentration of LpAE, LpEE, and LpHE was assayed in triplicates, the absorbance of untreated cells was considered as 100%, and the cytotoxic dose that kills 50% of cells population (IC_50_) was determined from the absorbance versus concentration curve. The above-mentioned method also was used to determine the IC_50_ value of *L. pumila* active fraction.

### 2.7. Cell Cycle Analysis by Flow Cytometry

The effects of *L. pumila* on cell proliferation was further studied using its active fraction. In order to determine the effect of *L. pumila* on the cell cycle, flow cytometry analysis was carried out. For this purpose, cells were seeded in 6-well plates at a density of 1 × 10^5^ cells/mL for 24 h. After incubation, cells were treated with different concentrations of the active fraction based on its IC_50_ value. Floating and attached cells were harvested, rinsed in PBS, fixed in ice-cold ethanol (70% v/v), and kept at −20°C for 30 minutes. After incubation, the mixture was centrifuged for 5 min at 600 ×g at 4°C, and the resultant pellet was further treated with DNase-RNase A at 2 mg/mL for 30 min. The cell pellet was then stained with propidium iodide (50 *μ*g/mL), containing 0.1% Triton X-100 and EDTA (0.02 mg/mL). Cell cycle profiles were determined by using *Cyan* software (Dako Cytomation, Germany).

### 2.8. Apoptosis Assay

The antiproliferative activity of *L. pumila* most active fraction was further investigated by apoptosis assay to determine the mode of cell death involved. For this purpose, treated and untreated cells were subjected to Giemsa staining, and the induction of apoptosis was further confirmed by acridine orange-ethidium bromide (AO-EB) double-staining.

For Giemsa staining, cells were seeded at 1 × 10^5^ cells/mL on glass slides for 24 h to allow cell adherence. After incubation, cells were then treated with the concentration of IC_50_ of *L. pumila* most active fraction for 24, 48, and 72 hours. At the end of the treatment, the cells were rinsed twice with cold PBS and further fixed with 100% cold methanol for 15 minutes. Cells were then stained with Giemsa for 30 minutes and rinsed under running tap water and air-dried before being observed under a light microscope.

As for the AO-EB staining, cells were seeded at 1 × 10^5^ cells/mL in 6-well plates for 24 h to allow cell adherence. After incubation, cells were treated with the concentration of IC_50_ of *L. pumila* most active fraction for 24, 48, and 72 hours. At the end of the treatment, cells were trypsinized with 0.025% (w/v) trypsin solution and rinsed with PBS once. Twenty-five (25) *μ*L of the cell suspension was then mixed together with 1 *μ*L of AO-EB dye cocktail, containing 100 mg/mL of each dye. Cells were immediately visualized under a fluorescence microscope. The quantification of apoptotic cells was according to Ribble et al. [25], where apoptotic cells showed green or orange condensed or fragmented chromatin. At least 600 nuclei per pellet were scored using a fluorescence microscope at a magnification of 400x and the percentage of apoptotic cells were determined as follows [26]:

(1) $$\text{Apoptotic cells,}\;\%=\left(\frac{\text{apoptotic cell number}}{\text{total number}}\right)\times 100.$$

### 2.9. Western Blot Analysis

To determine the effect of *L. pumila* on protein expression of Bax, Bcl-2, p53, and caspases-9, -7, and -8 in HM3KO cells, cells were seeded in 6-well plates at a density of 1 × 10^5^ cells/mL for 24 h. After incubation, cells were treated with the concentration of IC_50_ value of *L. pumila* most active fraction for 3, 6, and 24 hours. After incubation of each indicated times, the cells were scrapped in extraction buffer containing 50 mM Tris-HCl (pH 8.0), 150 mM NaCl, 5 mM EDTA, 5 mM EGTA, 1% (v/v) Triton-X, 1 mM phenylmethylsulfonyl fluoride (PMSF), 10 mM glycerophosphate, 1 mM Na_3_VO_4_, 10 *μ*g/mL pepstatin A, 10 *μ*g/mL aprotinin, 20 *μ*g/mL leupeptin, and 5 mM DTT. The mixture was then put on ice for 45 minutes and further subjected to three freeze-thaw cycles and was then centrifuged at 10,000 rpm for 20 min at 4°C. The protein concentration was determined by DC Bio-Rad protein assay according to the manufacturer's instructions. For Western blot analysis, 30 ug of proteins from both treated and untreated cells was subjected to separation by using SDS-PAGE over 4–12% gradient gels. After electrophoresis, the separated proteins were blotted or transferred onto polyvinyl-difluoride (PVDF) membrane (PolyScreen, NEN Life Sciences, USA). The membrane was then dried and blocked in blocking buffer (5% nonfat dry milk in PBS-Tween (0.1%) for 1 h at room temperature and further incubated with appropriate primary antibody in blocking buffer overnight at 4°C. This was followed by incubation with the appropriate secondary antibody coupled to horseradish peroxidase (HRP). The resultant immunoreactive bands were detected by Renaissance Western Blot Chemiluminescence Reagent Plus (NEN, Perkin Elmer, USA), exposed on a Kodak OMAT X-ray film (Eastman Kodak), and further subjected to a densitometry analysis, which was performed using a GS 670 Imaging Densitometer with Molecular Analyst software (Bio-Rad, Hercules, USA). The membranes were reprobed with *β*-actin antibodies as an internal control and to ensure equal loading. Each Western blot shown is a representative of at least three independent experiments.

### 2.10. Statistical Analysis

All data were expressed as the means ± standard deviation (SD) of the values obtained from at least three replicates. Using analysis of variance (ANOVA), statistical significance was determined. Mean values with probability values of *P* < 0.05 were taken as statistically significant.

## 3. Results

### 3.1. Antiproliferative Activity of *L. pumila* Extracts and Its Active Fraction

The antiproliferative activity of *L. pumila* extracts was determined using a methylene blue assay, and the IC_50_ values obtained were used as a parameter for cytotoxicity. The IC_50_ is the concentration required for 50% inhibition of a population of targeted cells [27], and crude extracts with IC_50_ values lower than 30 *μ*g/mL are considered cytotoxic [28].

Table 1 shows the antiproliferative activity of *L. pumila* various extracts against human melanoma HM3KO and nonmalignant MDBK and Vero cell lines. The ethanol extract (LpEE) was found more active against HM3KO cells, with an IC_50_ values of 16.18 ± 0.94 *μ*g/mL as compared to LpAE and LpHE (66.41 ± 0.78 and 35.67 ± 0.66 *μ*g/mL, resp.). Interestingly, LpEE demonstrated a certain level of cytoselectivity towards nonmalignant MDBK and Vero cell lines, with higher IC_50_ values of 129.22 ± 1.37 and 76.74 ± 0.86 *μ*g/mL, respectively. Comparatively, dacarbazine, a neoplastic drug, was used as a positive control in this study. Dacarbazine is widely used to treat malignant melanoma. In this study, dacarbazine exhibited an inhibition against HM3KO cells with an IC_50_ value of 5.32 ± 0.71 *μ*g/mL. (Table 1).

As the results from the antiproliferative assays revealed that ethanol extract of *L. pumila* (LpEE) was more cytotoxic against HM3KO cells as compared to aqueous (LpAE) and hexane (LpHE) extracts, thus LpEE was selected as the most active extract. In view of this, LpEE was then subjected to separation by using column chromatography and thin layer chromatography. From the column chromatography analyses, 131 fractions were collected and all of these fractions were then subjected to thin layer chromatography (TLC). Fractions with the same TLC profile were pooled and dried. Based on the TLC profiles obtained, these 131 fractions can be grouped into 9 major fractions (F1–F9). Of this nine fractions, F2 and F3 exhibited lower IC_50_ values of  9.3 ± 0.32 and 8.71 ± 0.41 *μ*g/mL, respectively. The rest of the fractions showed IC_50_ values of more than 90 *μ*g/mL. Even though F3 displayed values less than F2, F2 was chosen for further separation because the total yield of F2 was 7.4 times more (26.40 g) than F3 (3.58 g). From the chromatography analyses, F2 was successfully separated to 5 major fractions, namely, SF1–SF5. These fractions were then tested against HM3KO cells.  Table 2 shows the antiproliferative activity of *L. pumila* fractions (SF1–SF5) against HM3KO cells.

As shown in Table 2, fraction SF2 was found more effective against HM3KO cells with IC_50_ values of 7.59 ± 0.53 *μ*g/mL as compared to other fractions. Thus, this active fraction or designated as SF2Lp was then used to be further investigated in the apoptosis assay, cell cycle progression, and Western blot analyses.

### 3.2. Induction of Apoptosis by *L. pumila* Active Fraction, SF2Lp

Results from the antiproliferative assay suggested that fraction SF2 or termed as SF2Lp was the most active fraction with the IC_50_ values of  7.59 ± 0.53 *μ*g/mL as compared to other fractions. This value appeared to be lower than the IC_50_ value of active ethanol extract (16.18 ± 0.94 *μ*g/mL), indicating that SF2Lp is more cytotoxic to HM3KO cells than its crude extract. To determine the mode of cell death induced by *L. pumila* active fraction, SF2Lp in HM3KO cells, the cells were treated with SF2Lp at 7.59 *μ*g/mL in different time intervals (24, 48, and 72 hours).

**Figure 1:**
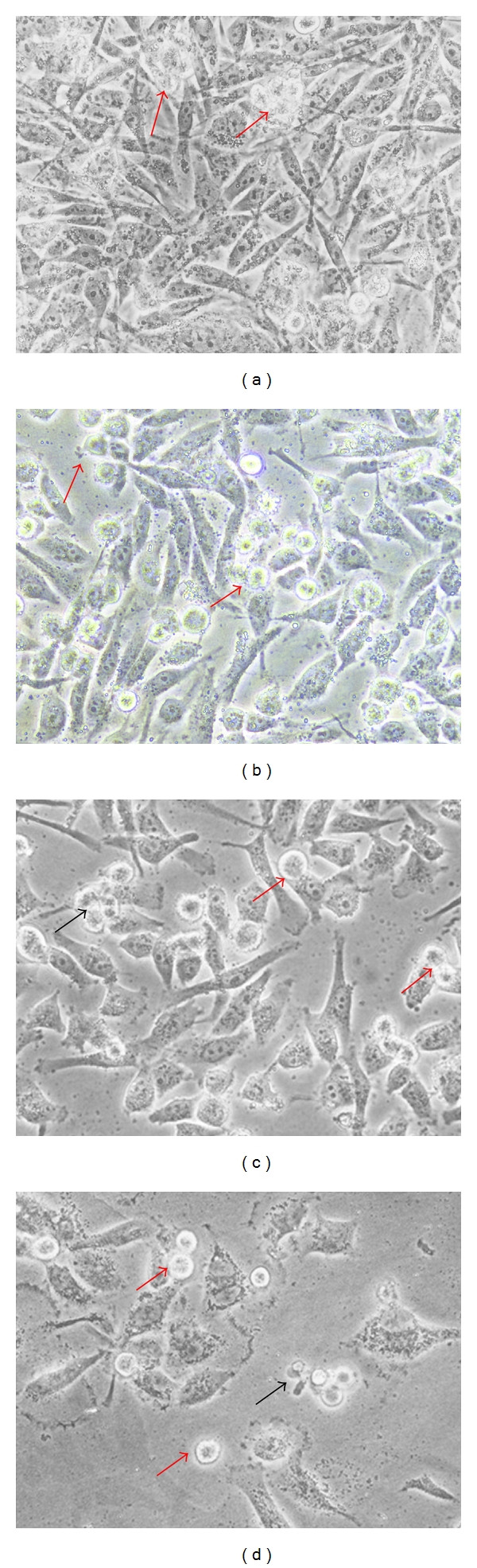
Representative images from morphological observation under phase contrast of an inverted microscope of SF2Lp-treated and -untreated HM3KO cells. HM3KO cells were treated with 7.59 *μ*g/mL SF2Lp for 24 (b), 48 (c), and 72 (d) hours. A population of 72 hours DMSO-treated HM3KO cells, which served as negative control, showed less apoptotic cells (a). Red arrows showed nuclear condensation and cells shrinkage due to apoptosis which occurred actively at the beginning of the treatment and the presence of apoptotic bodies (black arrows) after 72 hours of treatment. Magnification: 400x.

Under the phase contrast of an inverted microscope, the morphological changes of unstained HM3KO cells undergoing apoptosis can still be seen clearly (Figure 1). After 72-hour treatment, healthy HM3KO cells were seen as attached cells at the flask' surface, displaying regular epithelial-like shape and less number of apoptotic cells (Figure 1(a)), while SF2Lp-treated cells showed obvious morphological changes, including cell shrinkage and reduced in number. Some cells were spherical in shape indicating a degree of loss of attachment (Figures 1(b), 1(c), and 1(d)).

**Figure 2:**
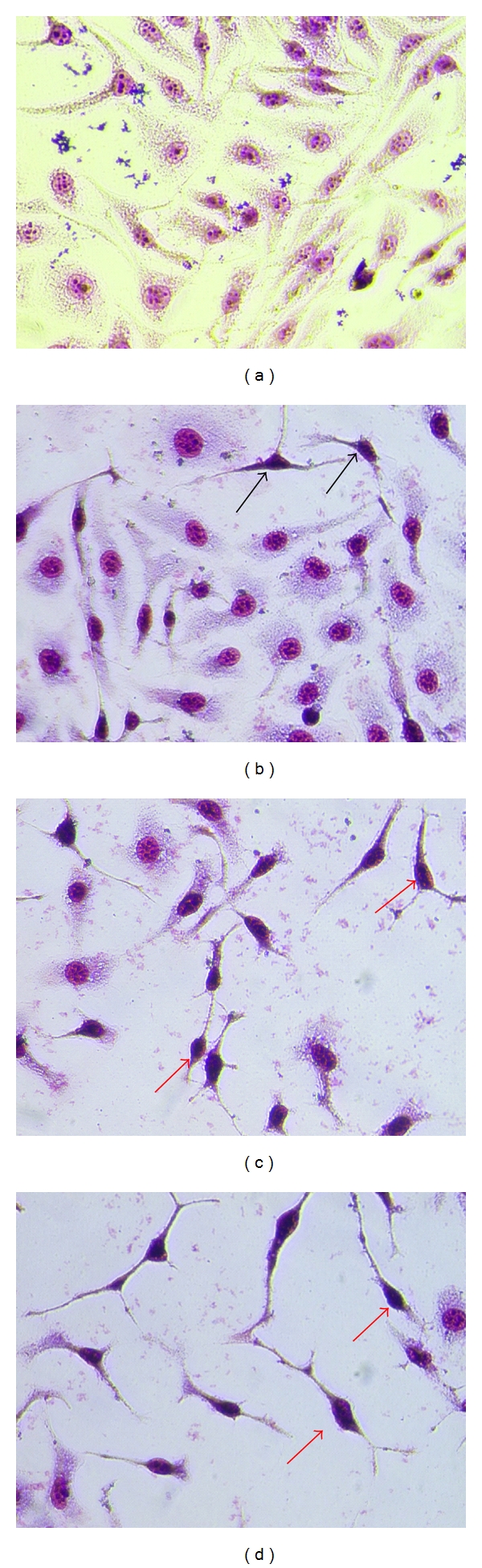
Representative images from morphological observation of SF2Lp-treated and -untreated HM3KO cells stained with Giemsa. HM3KO cells were treated with 7.59 *μ*g/mL SF2Lp for 24 (b), 48 (c), and 72 (d) hours. DMSO-treated HM3KO cells served as negative control (a). SF2Lp-treated cells showed significant morphological changes including shrinkage of cytoplasm and compaction of the nucleus (red arrow), indicating that cells underwent apoptosis. Magnification: 400x.

To detect the apoptotic cells, SF2Lp-treated cells were subjected to Giemsa staining, and the Giemsa stained-cells were observed under a light microscope. Microscopically, the healthy HM3KO cells displayed an ordinary epithelial-like shape with normal ratio of cytoplasm and nucleus (Figure 2(a)). In SF2Lp-treated cells, the morphological changes such as cytoplasmic condensation as well as highly condensed nucleus of cells undergoing apoptosis can be seen as early as 24-hour treatment. After 72-hour treatment the number of viable cells observed was significantly reduced as most of SF2Lp-treated cells were dead and sloughed off, leaving empty spaces behind (Figure 2(d)).

The inhibitory effect of SF2Lp on HM3KO cells via induction of apoptosis was further confirmed by using acridine orange (AO) and ethidium bromide (EB) nuclear staining. Acridine orange and ethidium bromide are two common fluorochromes that can be used to evaluate the nuclear morphology of apoptotic cells. It has been suggested that through this staining method, apoptotic index and cell membrane integrity can be assessed simultaneously, and as there is no cell fixation step, a number of potential artifacts thus can be avoided. In addition, fluorescence light microscopy together with AO-EB staining is a method of choice for its accuracy, simplicity, and rapidity [29]. While AO permeates both live and dead cells and makes the nuclei visibly green, EB only stains cells when their cytoplasmic membrane integrity is lost or compromised and, therefore, stains the nucleus red [29, 30]. By means of AO-EB staining assay, live cells exhibit normal green nuclei early apoptotic cells show bright green nuclei with condensed or fragmented chromatin, late apoptotic cells display condensed and fragmented orange chromatin, whereas necrotic cells showed structurally normal orange nucleus [29].

In this study, HM3KO cells were treated with SF2Lp at the concentration of its IC_50_ values of 7.59 *μ*g/mL for 24, 48, and 72 hours. HM3KO cells treated with 1% DMSO were used as control. Cells that showed bright green or orange condensed or fragmented chromatin were taken as apoptotic [25].

**Figure 3:**
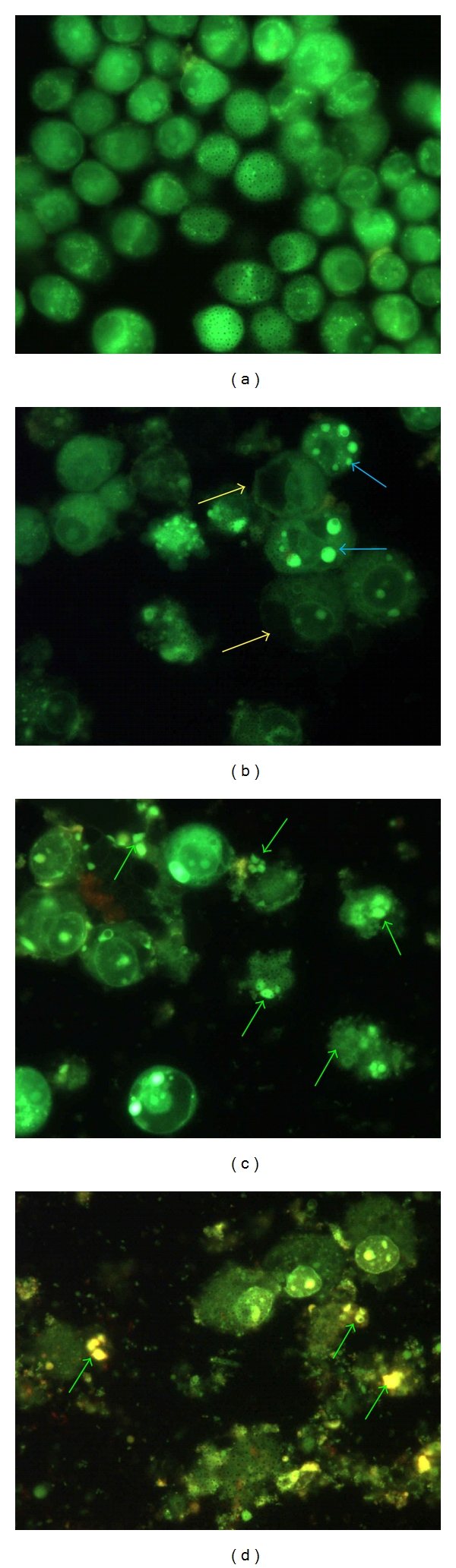
Representative images from morphological observation of SF2Lp-treated and -untreated HM3KO cells stained with acridine orange-ethidium bromide (AO-EB). HM3KO cells were treated with 7.59 *μ*g/mL SF2Lp for 24 (b), 48 (c), and 72 (d) hours. DMSO-treated HM3KO cells served as negative control (a). SF2Lp-treated cells showed significant morphological changes including nuclear condensation (blue arrows), membrane blebbing (yellow arrows), and apoptotic bodies (green arrows). Magnification for (a): 400x; (b)–(d): 1000x.

**Figure 4:**
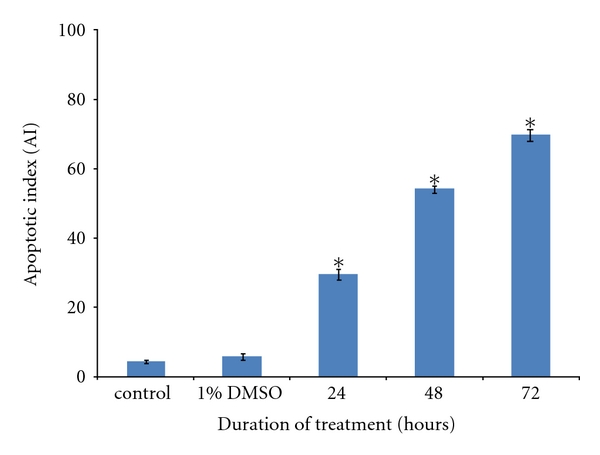
Induction of apoptosis in HM3KO cells by SF2Lp at the IC_50_ concentration (7.59 *μ*g/mL) at different exposure times (24, 48, and 72 h) as detected by AO-EB double staining. *P < 0.05 was taken as significantly different from control. Each value represents means ± SD from three independent experiments. SF2Lp-induced HM3KO cell death via apoptosis increased significantly in a time-dependent manner as compared to control.

Findings from the microscopic examination showed that live HM3KO cells in the control group displayed normal green nuclei (Figure 3(a)), while SF2Lp-treated cells showed membrane blebbing and bright dense granular masses of chromatin aggregated along the periphery of the nuclear membrane, indicating early apoptosis (Figure 3(b), blue arrow). In some of the treated cells, the nuclei were found disintegrated and fragmented into distinct spherical fragments with highly densed chromatin, suggesting the formation of apoptotic bodies in late apoptosis (Figures 3(c) and 3(d), green arrow). The percentage of apoptotic HM3KO cells for each exposure time (24, 48, and 72 hours) to SF2Lp were calculated and the data were presented as Apoptotic Index (AI) as displayed in Figure 4.

The apoptotic index (AI) was determined to confirm that SF2Lp-treated cell death was through apoptosis and it was calculated as the percentage of apoptotic cells from at least 600 counted cells within the cells population [26]. Apoptotic Index (AI) can be defined as the percentage of apoptotic cells and apoptotic bodies within the overall population of total cells [26]. The differences between the control group and treated group (1% DMSO, 24 hours, 48 hours, and 72 hours) were statistically analyzed using ANOVA, where *P* values <0.05 were considered as significant.

As shown in Figure 4, results from the study showed that the percentage of apoptotic HM3KO cells that have been treated with 7.59 *μ*g/mL of *L. pumila* active fraction, SF2Lp, were increased in a time-dependent manner with 29.5 ± 1.6% at 24 hours, 54.1 ± 1.1% at 48 hours, and 69.8 ± 1.7% at 72 hours. Untreated HM3KO cells that were cultured in enriched DMEM showed that only 4.3 ± 0.4% of these cell were apoptotic. On the other hand, cells that have been treated with 1% DMSO as negative control displayed only 5.7 ± 0.9% apoptotic cells and this value was not significantly different (*P* < 0.05) as compared to untreated control cells. This observation indicated that longer the exposure time to SF2Lp, the higher the percentage of apoptotic HM3KO cells.

### 3.3. Active Fraction of *L. pumila*, SF2Lp-Induced Cell Cycle Arrest at G1 Phase in HM3KO Cells

Cell cycle analyses were performed to investigate the basis of antiproliferative activity of *L. pumila* active fraction, SF2Lp, in HM3KO cells by flow cytometry analysis, and for this purpose, cells were treated with 3.00, 7.59, and 15.00 *μ*g/mL of SF2Lp for 24 hours. The concentrations were chosen based on the IC_50_ values of SF2Lp against HM3KO cells. The G1/S ratio was used as an index of G1 arrest [31].

**Figure 5:**
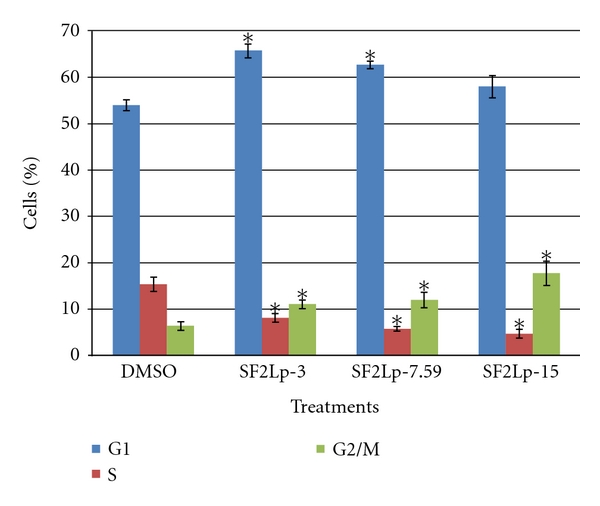
Cell cycle distribution in HM3KO cells treated with SF2Lp at 3 *μ*g/mL (SF2LP-3), 7.59 *μ*g/mL (SF2LP-7.59), and 15 *μ*g/mL (SF2LP-15) for 24 hours. DMSO-treated cells were used as control. The data represent the mean ± SD of 3 independent experiments. *Significantly different at *P* < 0.05 when the treated group was compared with the control.

**Figure 6:**
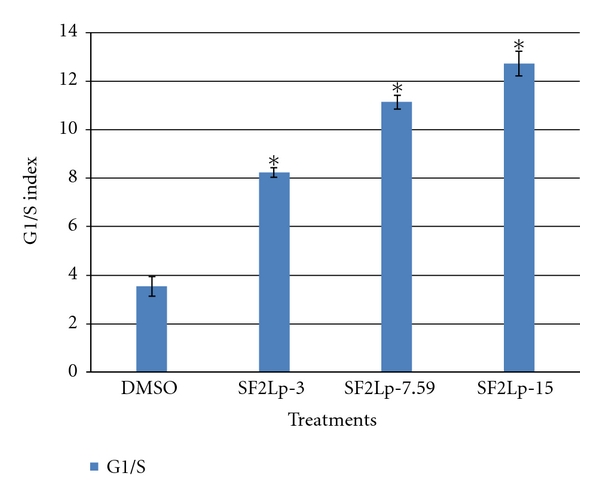
The G1/S index in growing HM3KO cells treated with SF2Lp at 3 *μ*g/mL (SF2LP-3), 7.59 *μ*g/mL (SF2LP-7.59), and 15 *μ*g/mL (SF2LP-15) for 24 hours. DMSO-treated cells were used as control. The data represent the mean ± SD of 3 independent experiments. *Significantly different at *P* < 0.05 when the treated group was compared with the control.

As shown in Figure 5, after 24 hours treatment, at the concentration of 3, 7.59, and 15 *μ*g/mL of SF2Lp, the percentage of cells in G1 phase reached to 65.58 ± 1.5, 62.52 ± 0.8, and 57.83 ± 1.4%, respectively, of total cells when compared to 53.85 ± 1.1% in the control group (*P* < 0.05). In addition, the percentage of cells in the S phase were significantly (*P* < 0.05) declined from 15.22 ± 1.0% in the control group to 7.97 ± 0.9, 5.61 ± 0.5, and 4.54 ± 0.5%, in the SF2Lp-3, SF2Lp-7.59, and SF2Lp-15 group, respectively, with a slight increase from 6.25 ± 0.9 in the control group to 10.93 ± 0.7, 11.87 ± 0.7, and 17.63 ± 0.5% in the SF2Lp-3, SF2Lp-7.59, and SF2Lp-15 group, respectively, of the G2/M phase cells (*P* < 0.05). After a 24-hour exposure to SF2Lp, the G1/S ratio of the treatment group was significantly higher than that of the control group (*P* < 0.05). A constant increasing pattern of the G1/S index was observed in the treatment group while the G1/S ratio of the control group was decreasing concomitantly through 10–24 h treatment. After a 24-hour exposure to SF2Lp, the G1/S index of HM3KO cells was 2.3 to 3.6 times higher than that of the control group (Figure 6).

These observations clearly suggest that the active fraction of  *L. pumila*, SF2Lp, was able to alter the cell cycle distribution of the growing HM3KO cells in a dose-dependent manner. In addition, after 24 hours of treatment, SF2Lp had caused a significant increased in cell numbers in G_1_ phase together with a marked reduction of the cell populations in S phase indicating that SF2Lp can effectively suppress HM3KO cells growth and proliferation by arresting the cell cycle at G1/S transition phase. 

### 3.4. *L. pumila* Active Fraction, SF2Lp, Induced Expression of p53 and Its Downstream Regulator Bax and Suppressed the Antiapoptotic Bcl-2 Expression

The p53 protein plays a vital role in apoptosis and lack of its expression or function may increased the risk of tumor formation [32]. To determine the role of p53 in the induction of apoptosis in HM3KO cells by SF2Lp, the total p53 protein levels was assessed by Western blot analysis.

**Figure 7:**
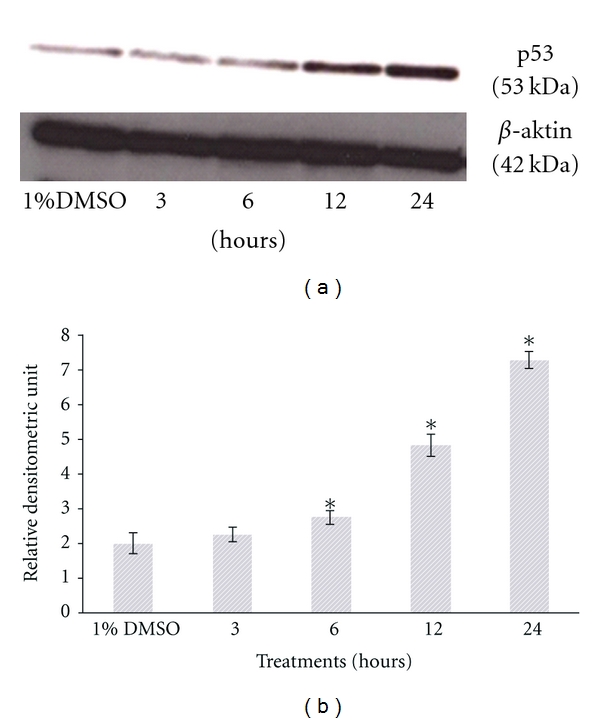
Representative Western blots showing the effect of SF2Lp at its IC_50_ concentration (7.59 *μ*g/mL) on expression level of p53 in HM3KO cells at different time intervals (3, 6, 12, and 24 hours). To confirm equal loading, the membrane was reprobed with *β*-actin. The data represent the mean ± SD of 3 independent experiments. *Significantly different at *P* < 0.05 over control group.

In this study, the results obtained showed that the expression level of p53 in SF2Lp-treated HM3KO cells was significantly increased (*P* < 0.05) after 6 hours of exposure with SF2Lp at its IC_50_ concentration (7.59 *μ*g/mL) and the level was found maximum after 24-hour treatment with SF2Lp as compared to control (Figure 6). This observation suggests that SF2Lp-induced apoptosis of HM3KO cells could be mediated through a p53-dependent pathway (Figure 7).

**Figure 8:**
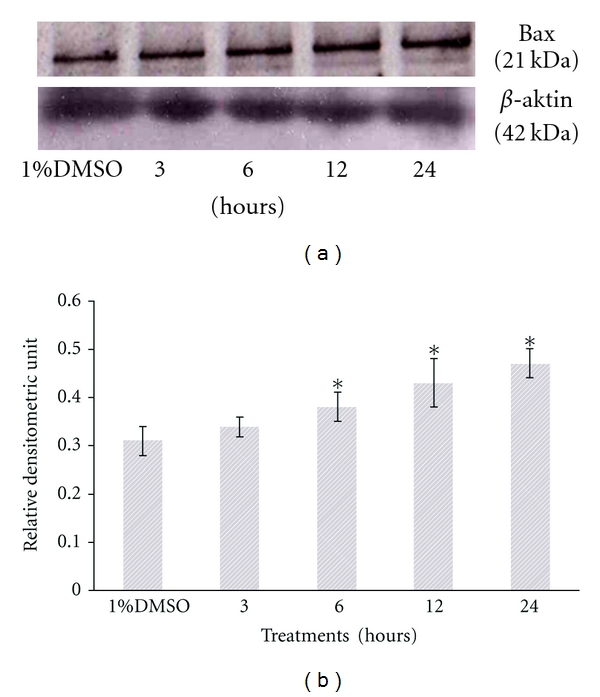
Representative Western blots showing the effect of SF2Lp at its IC_50_ concentration (7.59 *μ*g/mL) on the expression level of proapoptotic Bax in HM3KO cells at different time intervals (3, 6, 12, and 24 hours). To confirm equal loading, the membrane was reprobed with *β*-actin. The data represent the mean ± SD of 3 independent experiments. *Significantly different at *P* < 0.05 over control group.

**Figure 9:**
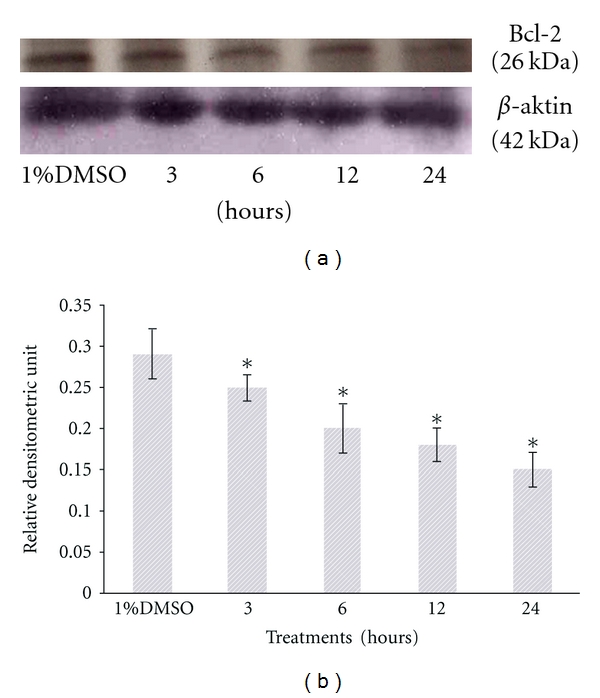
Representative Western blots showing the effect of SF2Lp at its IC_50_ concentration (7.59 *μ*g/mL) on the expression level of antiapoptotic Bcl-2 in HM3KO cells at different time intervals (3, 6, 12, and 24 hours). To confirm equal loading, the membrane was reprobed with *β*-actin. The data represent the mean ± SD of 3 independent experiments. *Significantly different at *P* < 0.05 over control group.

**Figure 10:**
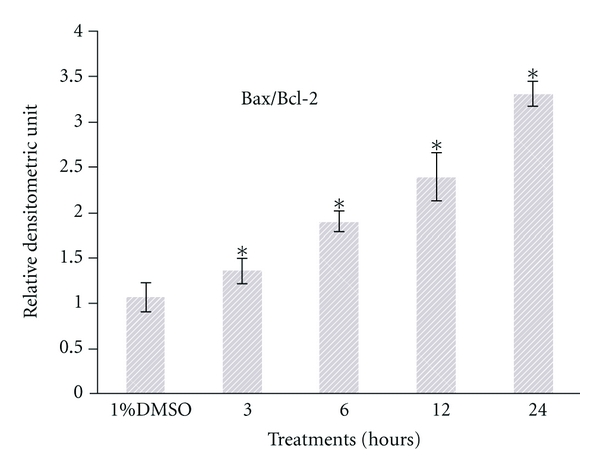
Effects of *L. pumila* active fraction, SF2Lp, at is IC_50_ concentration (7.59 *μ*g/mL) on the Bax/Bcl-2 ratio in HM3KO cells at different time intervals (3, 6, 12, and 24 hours). The data represent the mean ±  SD of 3 independent experiments. *Significantly different at *P* < 0.05 over control group.

In the next part of the experiment, results from the Western blot analysis showed that SF2Lp treatment at its IC_50_ concentration (7.59 *μ*g/mL) was able to concurrently increase the expression level of proapoptotic protein Bax and reduce the expression level of antiapoptotic protein BCl-2 in HM3KO cells in a time-dependent manner (Figures 8 and 9), directly contributing to the increase in Bax/Bcl-2 ratio (Figure 10). As shown in Figure 8, after 6 hours of treatment, SF2Lp was found to be able to increase the Bax protein levels in HM3KO cells treated with 7.59 *μ*g/mL of SF2Lp, while the antiapoptotic Bcl-2 protein levels were found decreased and this was evident after 12 hours of treatment with SF2Lp. According to Cory et al. [33], the susceptibility of tumor cells to the induction of apoptosis by chemotherapeutic agents is controlled by the ratio of Bcl-2/Bax proteins in the mitochondria. Thus, these findings suggest that the increased Bax/Bcl-2 ratio together with a marked increase in the level of p53 protein expression, in part, may contribute to the induction of apoptosis in SF2Lp-treated HM3KO cells through a p53-dependent apoptotic pathway.

## 4. Discussion

The objective of this study was to evaluate the antiproliferative effect of *L. pumila* extracts in HM3KO cells proliferation and to elucidate the possible molecular mechanisms that may occur as a result of exposing HM3KO cells with SF2Lp.

Both cell proliferation and apoptotic cell death are important determinants of growth of a tumour [5]. A balance between the two is critical in maintaining tissues homeostasis and normal development. As many chemotherapeutic agents have been identified to be able to induce apoptosis in cancer cells [34–36], apoptosis has been considered as a method for the treatment of cancer. Altered apoptosis, however, may contribute to development of cancer and other primary human diseases such as autoimmune diseases and neurodegenerative disorders [8, 37, 38]. Observation of morphological changes as cells undergo apoptosis still remains the most reliable technique to define apoptotic cell death [39]. Even though light microscopy has a low capacity to distinguish apoptotic cells, the acuity can be enhanced by using nuclear fluorescent dyes to examine nuclear changes such as compacted chromatin and fragmented DNA.

This study has demonstrated that the treatment of HM3KO cells with *L. pumila* extracts resulted in an inhibition of cell proliferation and a concomitant decrease in cell viability. Results obtained from the study suggested that among the various (aqueous-LpAE, ethanolic-LpEE, and hexane-LpHE) *L. pumila* extracts tested, LpEE showed the highest antiproliferative activity against HM3KO cells. Interestingly, the cytotoxicity of LpEE appeared to be higher against HM3KO cells than on nonmalignant MDBK and Vero cell lines. The 50% inhibitory concentration (IC_50_) of LpEE against HM3KO cells was 16.18 ± 0.94 *μ*g/mL, while its active fraction, SF2Lp, showed lower values that is 7.59 ± 0.  0.53 *μ*g/mL. From this point onwards, SF2Lp was used to further elucidate the molecular mechanism involved in SF2Lp-induced HM3KO cells apoptosis. Findings from the antiproliferative and apoptosis assays suggest that SF2Lp was able to induced cell death of HM3KO cells.

To confirm that SF2Lp-treated cell death was through apoptosis, the extent of cell death was investigated by using nuclear staining assays and Apoptotic Index (AI) was then calculated. AI can be defined as the percentage of apoptotic cells and apoptotic bodies within the overall population of total cells [26].

Results from the Giemsa staining assays clearly show the morphology of apoptotic HM3KO cells and the apoptotic cells can be seen only after 24 hours of treatment (Figure 2).

The ability of SF2Lp in inducing apoptotic cell death in HM3KO cells was confirmed by the acridine orange-ethidium bromide (AO-EB) nuclear staining assay that displayed presence of obvious changes associated with apoptosis, which include cell shrinkage, condensation of nuclear chromatin, and apoptotic bodies in SF2Lp-treated cells. The Apoptotic Index (AI) calculated from AO-EB assay further revealed that the percentage of apoptotic HM3KO cells that have been treated with 7.59 *μ*g/mL of *L. pumila* active fraction, SF2Lp, were increased in a time-dependent manner with 29.5 ± 1.6% at 24 hours, 54.1 ± 1.1% at 48 hours, and 69.8 ± 1.7% at 72 hours. This observation indicated that the longer the exposure time to SF2Lp, the higher the percentage of apoptotic HM3KO cells. These findings suggest that antiproliferative effect of SF2Lp in HM3KO cells was via induction of apoptosis and that the active components present in SF2Lp have the ability to induce cancer cell death *in vitro.*

Many anticancer molecules show growth inhibition and/or apoptotic cell death of cancer cells by modulating the cell-cycle regulatory molecules [40]. To find out the mechanism of action of SF2Lp in inhibiting the HM3KO cells' growth, the effects of SF2Lp on the cell cycle and its ability to induce apoptosis in HM3KO cells were studied. Results obtained from the flow cytometry analysis showed that SF2Lp was able to arrest the cell cycle at the G1 phase to prevent the HM3KO cells transition from G1 to S phase. The ability of SF2Lp to arrest the cell cycle and to induce the apoptosis process in HM3KO cells is believed to contribute to the antiproliferative activity of SF2Lp.

In order to examine the mechanism of action of SF2Lp in inducing apoptosis in HM3KO cells at the molecular level, Western blotting analysis was thus carried out. Results from the mechanistic study showed that SF2Lp was able to concurrently increase the expression level of proapoptotic protein Bax and reduce the expression level of antiapoptotic protein in HM3KO cells. This phenomenon is directly contributing to the increase in Bax/Bcl-2 ratio that drives cells to undergo apoptosis [33]. To find out whether apoptosis induction by SF2Lp involves the tumor suppressor protein p53, the effect of SF2Lp on the expression level of p53 was also studied. Results from the Western blotting analysis showed that SF2Lp was able to increase the expression level of p53 in HM3KO cells. These findings suggest that the ability of SF2Lp to arrest the cell cycle at G1 phase and to induce the apoptosis process in HM3KO cells was possibly mediated by the activity of p53.

All in all, the above-mentioned findings suggest that the increased Bax/Bcl-2 ratio together with a marked increase in the level of p53 protein expression, in part, may contribute to the antiproliferative activity and induction of apoptosis in SF2Lp-treated HM3KO cells through a p53-dependent apoptotic pathway.

There are very limited numbers of scientific papers published regarding the inhibitory effects of *L. pumila* towards cell proliferation as well as apoptosis induction in cultured cells. To the best of our knowledge, the present study is the first to report the antiproliferative and proapoptotic effects of *L. pumila* ethanol extract and its active fraction, SF2Lp, in human melanoma HM3KO cells *in vitro*.

## 5. Conclusion

Altogether the results from this study, showed antiproliferative effects of *L. pumila* active fraction, SF2Lp, through the regulation of the cell cycle progression and the expression of proteins involved in apoptotic pathway in HM3KO cells. From this study we can conclude that *L. pumila* was able to inhibit HM3KO cell growth possibly by arresting the cell cycle at G1 phase and inducing apoptosis in HM3KO cells via the up- and down-regulation of Bax/Bcl-2 protein, which mediated through a p53-dependent pathway.

## Figures and Tables

**Table 1:** Antiproliferative activity of *L. pumila* various extracts in human melanoma HM3KO and nonmalignant MDBK and Vero cell lines.

* L. pumila *extracts	IC_50_ values (*μ*g/mL)		
	HM3KO	MDBK	Vero
LpAE	66.41 ± 0.78	89.30 ± 1.95	107.62 ± 0.98
LpEE	16.18 ± 0.94	129.22 ± 1.37	76.74 ± 0.86
LpHE	35.67 ± 0.66	57.90 ± 1.09	36.78 ± 1.63
Dacarbazine	5.32 ± 0.71	39.52 ± 0.76	54.11 ± 0.56

All values were expressed as the means ±  SD from three independent experiments: *L. pumila* aqueous extract-LpAE, ethanol extract-LpEE, and hexane extract-LpHE.

**Table 2:** Antiproliferative activity of *L. pumila* various fractions (SF1–SF5) against human melanoma HM3KO cells.

* L. pumila* fractions	IC_50_ values (*μ*g/mL)
SF1	35.80 ± 0.27
SF2	7.59 ± 0.53
SF3	47.51 ± 0.87
SF4	63.88 ± 0.59
SF5	93.91 ± 0.68

All values were expressed as the means ± SD from three independent experiments.

## References

[1] Lee HJ, Lee EO, Rhee YH (2006). An oriental herbal cocktail, ka-mi-kae-kyuk-tang, exerts anti-cancer activities by targeting angiogenesis, apoptosis and metastasis. *Carcinogenesis*.

[2] Issa AY, Volate SR, Wargovich MJ (2006). The role of phytochemicals in inhibition of cancer and inflammation: new directions and perspectives. *Journal of Food Composition and Analysis*.

[3] Cragg GM, Newman DJ (2005). Plants as a source of anti-cancer agents. *Journal of Ethnopharmacology*.

[4] Jordan MA, Wilson L (2004). Microtubules as a target for anticancer drugs. *Nature Reviews Cancer*.

[5] Lowe SW, Lin AW (2000). Apoptosis in cancer. *Carcinogenesis*.

[6] Carson DA, Ribeiro JM (1993). Apoptosis and disease. *The Lancet*.

[7] Fisher DE (1994). Apoptosis in cancer therapy: crossing the threshold. *Cell*.

[8] Thompson CB (1995). Apoptosis in the pathogenesis and treatment of disease. *Science*.

[9] Gosslau A, Chen KY (2004). Nutraceuticals, apoptosis, and disease prevention. *Nutrition*.

[10] Böhm I, Schild H (2003). Apoptosis: the complex scenario for a silent cell death. *Molecular Imaging and Biology*.

[11] Call JA, Eckhardt SG, Camidge DR (2008). Targeted manipulation of apoptosis in cancer treatment. *The Lancet Oncology*.

[12] Gerl R, Vaux DL (2005). Apoptosis in the development and treatment of cancer. *Carcinogenesis*.

[13] Srinivas K (2010). Anticancer and antimicrobial activity of embelin derivatives. International. *Journal of Preclinical and Pharmaceutical Research*.

[14] Zheng ZF, Xu JF, Feng ZM, Zhang PC (2008). Cytotoxic triterpenoid saponins from the roots of *Ardisia crenata*. *Journal of Asian Natural Products Research*.

[15] Li M, Wei SY, Xu B (2008). Pro-apoptotic and microtubule-disassembly effects of ardisiacrispin (A+B), triterpenoid saponins from *Ardisia crenata* on human hepatoma Bel-7402 cells. *Journal of Asian Natural Products Research*.

[16] Zakaria M, Mohd MA (1994). *Traditional Malay Medicinal Plants*.

[17] Burkill IH (1993). *A Dictionary of the Economic Products of the Malay Peninsula*.

[18] Stone BC (1998). Notes on the genus *Labisia* Lindl. (Myrsinaceae). *Malayan Nature Journal*.

[19] Husniza H (2002). *Estrogenic and Androgenic Activities of Kacip Fatimah (Labisia pumila)*.

[20] Pandey A, Kour K, Bani S (2008). Effects of aqueous extract of *Labisia pumila* on immune profile of pregnant rats. *Journal of Tropical Medicinal Plants*.

[21] Choi H-K, Kim D-H, Kim JW, Ngadiran S, Sarmidi MR, Park CS (2010). *Labisia pumila* extract protects skin cells from photoaging caused by UVB irradiation. *Journal of Bioscience and Bioengineering*.

[22] Kamuhabwa A, Nshimo C, De Witte P (2000). Cytotoxicity of some medicinal plant extracts used in Tanzanian traditional medicine. *Journal of Ethnopharmacology*.

[23] Mulder NH, Van der Graaf WTA, Willemse PHB, Schraffordt Koops H, De Vries EGE, Sleijfer TD (1994). Dacarbazine (DTIC)-based chemotherapy or chemoimmunotherapy of patients with disseminated malignant melanoma. *British Journal of Cancer*.

[24] Lin L, Hwang PL (1991). Antiproliferative effects of oxygenated sterols: positive correlation with binding affinities for the antiestrogen-binding sites. *Biochimica et Biophysica Acta*.

[25] Ribble D, Goldstein NB, Norris DA, Shellman YG (2005). A simple technique for quantifying apoptosis in 96-well plates. *BMC Biotechnology*.

[26] Soini Y, Paakko P, Lehto VP (1998). Histopathological evaluation of apoptosis in cancer. *American Journal of Pathology*.

[27] Cheng HC (2001). The power issue: determination of K_B_ or K_i_ from IC_50_—a closer look at the Cheng-Prusoff equation, the Schild plot and related power equations. *Journal of Pharmacological and Toxicological Methods*.

[28] Suffness M, Pezzuto JM, Hostettmann K (1990). Assays related to cancer drug discovery. *Methods in Plant Biochemistry: Assay for Bioactivity*.

[29] Renvoize C, Biola A, Pallardy M, Breard J (1998). Apoptosis: identification of dying cells. *Cell Biology and Toxicology*.

[30] Jayadev R, Jagan MRP, Malisetty VS, Chinthapally VR (2004). Diosgenin, a steroid saponin of *Trigonella foenum graecum* (Fenugreek), inhibits azoxymethane-induced aberrant crypt foci formation in F344 rats and induces apoptosis in HT-29 human colon cancer cells. *Cancer Epidemiology Biomarkers and Prevention*.

[31] Sun J, Hai Liu R (2006). Cranberry phytochemical extracts induce cell cycle arrest and apoptosis in human MCF-7 breast cancer cells. *Cancer letters*.

[32] Clarke AR, Gledhill S, Hooper ML, Bird CC, Wyllie AH (1994). p53 dependence of early apoptotic and proliferative responses within the mouse intestinal epithelium following *γ*-irradiation. *Oncogene*.

[33] Cory S, Huang DCS, Adams JM (2003). The Bcl-2 family: roles in cell survival and oncogenesis. *Oncogene*.

[34] Liu J, Shen HM, Ong CN (2000). *Salvia miltiorrhiza* inhibits cell growth and induces apoptosis in human hepatoma HepG_2_ cells. *Cancer Letters*.

[35] Hannun YA (1997). Apoptosis and the dilemma of cancer chemotherapy. *Blood*.

[36] Simstein R, Burow M, Parker A, Weldon C, Beckman B (2003). Apoptosis, chemoresistance, and breast cancer: insights from the MCF-7 cell model system. *Experimental Biology and Medicine*.

[37] Deng LY, Zhang YH, Xu P, Yang SM, Yuan XB (1999). Expression of interleukin 1*β* converting enzyme in 5-FU induced apoptosis in esophageal carcinoma cells. *World Journal of Gastroenterology*.

[38] Bani-Hani KE, Almasri NM, Khader YS, Sheyab FM, Karam HN (2005). Combined evaluation of expressions of cyclin E and p53 proteins as prognostic factors for patients with gastric cancer. *Clinical Cancer Research*.

[39] Huerta S, Goulet EJ, Huerta-Yepez S, Livingston EH (2007). Screening and detection of apoptosis. *Journal of Surgical Research*.

[40] Hahn WC, Weinberg RA (2002). Modelling the molecular circuitry of cancer. *Nature Reviews Cancer*.

